# [Expression of Concern] Long non-coding RNA BX357664 inhibits cell proliferation and metastasis in human lung cancer

**DOI:** 10.3892/ol.2026.15602

**Published:** 2026-04-16

**Authors:** Shu-Hui Xiao, Gong-Xiang Li, Lingli Quan

Oncol Lett 17: 2607–2614, 2019; DOI: 10.3892/ol.2019.9886

Following the publication of the above paper, and a Corrigendum that was published in 2021 to take account of changes in the authorship on the paper (doi: 10.3892/ol.2021.12436), it has been drawn to the Editor's attention by a concerned reader that, regarding the Transwell migration assay experiments shown in Fig. 4A on p. 2612, the A549/Control and 95 D/Vector, and A549/Vector and 95 D/Control, pairings of data panels respectively contained overlapping sections, such that data which were intended to show the results from differently performed experiments had apparently been derived from a smaller number of original sources.

The authors have been contacted by the Editorial Office to offer an explanation for these apparent anomalies in the presentation of the data in this paper, and we are awaiting their response. Owing to the fact that the Editorial Office has been made aware of potential issues surrounding the scientific integrity of this paper, we are issuing an Expression of Concern to notify readers of these potential problems while the Editorial Office continues to investigate this matter further.

